# Application of ultrasound technique to evaluate the testicular function and its correlation to the sperm quality after different collection frequency in rams

**DOI:** 10.3389/fvets.2022.1035036

**Published:** 2022-11-25

**Authors:** Rafael Montes-Garrido, Marta F. Riesco, Luis Anel-Lopez, Marta Neila-Montero, Cristina Palacin-Martinez, Juan Carlos Boixo, Paulino de Paz, Cristina Ortega-Ferrusola, Mohamed A. A. Hassan, Luis Anel, Mercedes Alvarez

**Affiliations:** ^1^Investigación en Técnicas de Reproducción Asistida – Universidad de León, Instituto de Desarrollo Ganadero y Sanidad Animal, University of León, León, Spain; ^2^Animal Reproduction and Obstetrics, Department of Veterinary Medicine, Surgery and Anatomy, University of León, León, Spain; ^3^Celular Biology, Department of Molecular Biology, University of León, León, Spain; ^4^Anatomy, Department of Veterinary Medicine, Surgery and Anatomy, University of León, León, Spain; ^5^Laboratory of Equine Reproduction and Equine Spermatology, Veterinary Teaching Hospital, University of Extremadura, Cáceres, Spain; ^6^Department of Anatomy and Embryology, Faculty of Veterinary Medicine, Sohag University, Sohag, Egypt

**Keywords:** Doppler, fertility, ovine, semen collection frequency, sperm quality, ultrasonography

## Abstract

The frequency of semen collection is a crucial factor to consider in the rams performance inside breeding centers workout. To evaluate this factor, ram Breeding Soundness Evaluation could include sperm quality evaluation and new predictive and non-invasive tools such as ultrasound technique. In this work, an advanced ultrasonography technology, analyzing the testicular volume, echotexture, and vascular function, was used in three different frequencies of semen collection (abstinence frequency, AF; standard frequency, SF; and intensive frequency, IF). Semen samples were cooled (15°C, 6 h) and evaluated in terms of production, motility, viability, apoptosis, and content of reactive oxygen species. Correlation coefficients were calculated between ultrasonography measurements of echotexture and blood flow and sperm quality parameters. Our results showed an increase in the testicular echotexture when the frequency of semen collection was intensified. Doppler parameters (PSV, RI, PI, TABF) increased (*P* ≤ 0.05) when the frequency of semen collection was intensified. The sperm motility and functionality decreased in the samples of IF (*P* ≤ 0.05), evidencing the frequency of semen collection's influence. Moreover, moderate positive correlations were established among echotexture and different Doppler parameters with motility parameters in SF. Furthermore, the influence of abstinence days on AI success was analyzed in a field assay. The highest fertility rates were obtained when males had two to five abstinence days. To conclude, frequency of semen collection could be influenced in terms of semen quantity and sperm quality, showing changes in parenchyma echotexture and testicular vascularization. The standard semen collection frequency was the most adequate option. In addition, ultrasonography may be a predictive tool for estimating variations in the sperm quality of donor rams subjected to different frequencies of semen collection in reproduction centers.

## Introduction

Reproductive performance is the most important parameter affecting flock profitability (1), and the reproductive capacity of the rams plays a key role (2). MacLaren (3) suggests that 50% of the reproductive potential of a flock is provided by the ram (3). Testis evaluations are performed to assess the overall potential capacity of rams to serve and impregnate ewes, also known as the Breeding Soundness Evaluation (BSE) (2). A BSE may include anatomical and structural examinations and assessment for health status, body condition score (BCS), testicular measurements, sperm quality, and libido (4). In this context, sexual behavior and semen characteristics are the main parameters limiting male reproductive efficiency, and both of them are greatly influenced by the frequency of semen collection. Variations in ejaculation frequency induce changes in the sperm maturation process, sperm functional characteristics, ionic composition, and enzymatic activity of the seminal plasma (5, 6). In a previous study, Ollero et al. (5) documented that different periods of abstinence could affect sperm quality in terms of viability, motility, and acrosome integrity in the ovine species. To our knowledge, there are no studies in a large number of rams assessing the effect of semen collection frequency in male reproductive capacity, semen production, and quality employing innovative and predictive techniques.

Traditional methods, such as measurement of scrotal circumference and libido or palpation and manipulation of the genital organs, have been used to evaluate the potential reproductive capacity of rams (3, 7, 8). In recent years, several new tools, including ultrasonography, have been used to predict variations in semen characteristics and the ram's reproductive capacity in reproduction centers and flocks (9, 10). Specifically, ultrasonography is a non-invasive, non-ionizing, and non-damaging technique and an indispensable tool in reproductive clinics that provides real-time and sequential information on male reproductive performance (11, 12). The B-mode ultrasound has been used in different domestic animals species as a valid tool to estimate the testicular volume (13–15), estimate the parenchyma echotexture (16–18), and identify uncertain clinical findings, such as early stages of macroscopic pathological processes or monitoring changes in lesions (12). Color and Pulse Doppler ultrasonography has been employed to characterize and quantify testes' blood flow in different species, such as stallions (19) and dogs (20). This technology has also been used to evaluate scrotal disorders in dogs (14) and camelids (21) and correlate them with sperm quality. Several studies have assessed using B-mode ultrasound in physiological and pathological conditions (12, 22–24), and the relationship between the puberty and the changes in the echogenicity of the parenchyma (25–28) in ovine andrology. In addition, several studies on the use of Doppler ultrasonography for the evaluation of testicular blood flow in rams are available (9, 10, 26, 29) that consider the influence of this parameter in testicular function. Testicular blood flow is the main route through which all the required nutrients, oxygen, regulatory hormones, and secretory products are regulated and exchanged to and from the testes (30). In this respect, different studies have evidenced an association between testicular blood flow and sperm quality in several species such as humans (31), stallions (13, 32), or rams (9, 10), and used Doppler parameters to diagnose fertility rates in camelids (21) or dogs (14). Because of this, we hypothesize that semen collection frequency could be a crucial factor causing testicular changes in those parameters detected by ultrasound and in sperm quality.

Considering the importance of rams management in terms of performance within a breeding center, the first objective of this study is to investigate the effects of the frequency of semen collection on male reproductive performance including testicular function, and semen quantity and quality employing a multiparametric approach based on testicular morphometry, echotexture and vascularization, blood testosterone level, libido, and sperm motility and functionality parameters. Secondly, the study aims to approach the possible association between sperm quality parameters and ultrasonography measurements in the different semen collection frequencies. As a third aim, we study the direct consequences of different semen extraction frequencies in terms of sperm quality and fertility in a field assay.

## Materials and methods

### Animals

Twent-five sexually mature (age range 2–7 years) Assaf rams were used during the breeding season in the current study. All the rams were previously examined, and they did not have any disease. Animals were housed grouped (five animals per each group) in closed pens with access to an open area at the Animal Selection and Reproduction Center of the Junta de Castilla y León (CENSYRA) (Villaquilambre, León, Spain), where they were fed on a standard balanced diet. The current study was performed in accordance with the Guidelines of the European Union Council (2010/63/EU), following Spanish regulations (RD/1201/2005, abrogated by RD/53/2013) for the use of laboratory animals. All the experiments were approved by the Institutional Animal Care and Use Committee at the University of León (ÉTICA-ULE-013-2018).

### Experimental design

#### Experiment 1: Evaluation of different semen collection frequencies

**Figure 1:**
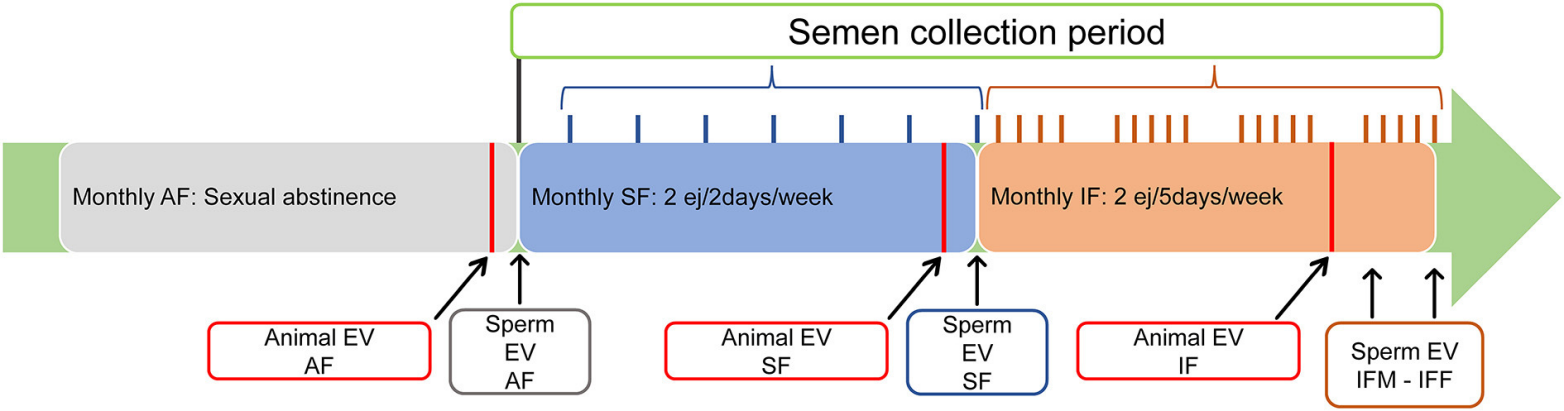
Experimental design. All three semen collection frequencies were extended for 1 month. Animal evaluation (Animal EV): clinical examination (inspection and palpation of external genitalia, libido, and blood collection) and ultrasonography evaluation (testicular volume, echotexture, and blood flow) were carried out at least 24 h before experimental semen collection). Sperm evaluation (Sperm EV): sperm production, motility, and physiology were performed at the end of the evaluated semen collection frequency. AF, abstinence semen collection frequency; SF, standard semen collection frequency; IFM, intensive semen collection frequency on Monday; IFF, intensive semen collection frequency on Friday.

Sexually mature Assaf rams were used during the breeding season. All rams were enrolled in the abstinence semen collection frequency (AF) where the males were sexually rested and semen was not collected for 1 month. At the end of this period, testicular volume, testicular echotexture, and Doppler parameters were assessed. After the animal's evaluation, two consecutive ejaculates per ram were collected and mixed, measuring the following parameters: ejaculate volume, sperm concentration, and total sperm production. Ejaculates were analyzed including motility and sperm physiology parameters (detailed in section “Sperm evaluation”). Then, all rams were enrolled in the standard semen collection frequency (SF) for 1 month: two consecutive ejaculates per day/two collection days per week. At the end of this period, all ultrasonographic measurements were repeated, and, after the animal's evaluation, two consecutive ejaculates per ram were collected, mixed, and analyzed. To conclude this experiment, the 25 rams were enrolled in the intensive semen collection frequency (IF): two consecutive ejaculates per day/five collection days per week (Monday–Friday). Again, 1 month later, all ultrasonographic measurements were repeated. In this scenario, after 2 days of abstinence by the weekend, two consecutive ejaculates per ram were collected and analyzed on Monday (IFM) and, after five consecutive days of semen collection, two consecutive ejaculates per ram were collected and analyzed on Friday (IFF). The experimental design is shown in Figure 1.

#### Experiment 2: Ram's performance and sperm yield: A field assay

In this part of the experiment, we tried to extrapolate our findings to field assay. In a reproduction center simulation, ejaculates obtained from our experimental rams (25 males) in experiment 1 were classified following the reproduction center criteria in valid (ejaculate volume > 0.5 ml, mass motility > 3, sperm concentration > 3,000 × 10^6^ sperm/ml) and non-valid ejaculates (some of the criteria below the minimum value) as previously described Neila-Montero et al. (33). Then, sperm quality, including motility and cytometry, was assessed in both experimental groups.

For the fertility trial, the experimental groups were classified by male abstinence period before semen collection for insemination: the fertility standard interval (SI), when seminal doses were obtained from males with two–five abstinence days before semen collection for AI; the fertility high interval (HI), when seminal doses were obtained from males with at least 10 abstinence days before semen collection for AI; the fertility low interval (LI), when seminal doses were obtained from males with semen collection the previous day for AI; the annual fertility (AN), annual average fertility from the National Association of Breeders of the Assaf Sheep Breed (ASSAF.E). A descriptive assay was performed to assess the effect of the abstinence period of semen collection on fertility AI.

For this purpose, 357 seminal doses (400 × 10^6^ sperm/dose) from 10 mature Assaf rams housed in the Sheep and Goat Selection and Genetic Improvement Center of Castilla y León (Ovigén; Villalazán, Junta de Castilla y León, Spain) were used during the breeding season. Ejaculates were diluted to 1,600 × 10^6^ sperm/ml with INRA 96 and cooled using a rate of −0.5°C/min from 30°C down to 15°C using a programmable water bath (CC-K8, Huber, Germany). A total of 357 Assaf adult ewes from six commercial farms were inseminated 6–8 h after semen collection. Females were subjected to treatment for estrus induction and synchronization using intravaginal sponges with 20 mg fluorogestone acetate (Chronogest^®^, MSD, Kenilworth, NJ, USA) over 14 days. The sponges were removed, and ewes were treated with 500 IU of eCG intramuscular (Folligon^®^, MSD, Bogotá, Colombia). Cervical artificial inseminations (54 ± 1 h post sponges removal) were performed by two experienced veterinarians of ASSAF.E. Reproductive success was evaluated in terms of fertility (lambing ewes/inseminated ewes (%) according to the births registered at 137–154 days post-insemination).

### Previous clinical examination

Before being included in the study, every single male underwent a general clinical examination, visual inspection of the scrotum, and testicular palpation for the evaluation of consistency, symmetry, mobility, and sensitivity of testes. Epididymis and pampiniform plexus were also palped in order to ensure that no observable gross pathology was resent on the external genitalia. Then, the rams were mildly sedated with xylazine (0.05 mg/kg), administered intramuscularly, and restrained in the standing position using a containment rack. The wool on both sides of the scrotum was shaved.

### Testosterone levels

After all clinical measures, blood samples were collected into a vacutainer tube without anticoagulant from the jugular vein. The samples were refrigerated at 5°C, and the blood serum was collected and stored at −20°C until assayed. A commercial ELISA kit using the Immulite 2000 XPi Immunoassay System (Siemens, Eschborn, Germany) was used to determine the serum testosterone. According to the manufacturer's instructions, the sensitivity was 0.15 ng/ml, and the intra- and inter-assay coefficients of variation were 5.1 and 7.2% when the average samples were 9.91 ng/ml.

### Libido evaluation

Libido was subjectively categorized (zero to 10 score). We calculated time spent from contact with a female decoy to ejaculation in both daily semen collections. Time intervals were used to qualify the rams. If ejaculation occurred in 1 min or less, the male obtained the maximum score (10). If ejaculation occurred in 1–2 min, the male obtained a score of nine. If ejaculation occurred in 2–3 min, the male obtained a score of eight, etc. When ejaculation occurred after more than 10 min, the male obtained a score of zero. The average score of the two ejaculations was calculated.

### Testicular volume

All the ultrasonographic measurements were carried out by the same technician. All examinations were performed using a real-time ultrasound scanner, EXAPAD (IMV, France), equipped with a 7.5 MHz linear array. The transducer was covered with a copious amount of gel to facilitate ultrasonographic imaging. Scanning was performed without pressure to avoid a distortion of the testicular shape. Images of the caudocranial, lateral-lateral, and ventrodorsal axis of the testes were obtained. The testicular width, height, and length were measured using electronic calipers integrated into the ultrasound machine. Cursors were set at the borders of the tunica albuginea. The measurements were performed in triplicate of three different images (technical replicates). The echogenicity, homogeneity, and surface of the scrotal contents were also assessed. The testicular volume was calculated using the formula described by Hedia et al. (9): *L* × *H* × *W* × 0.71.

### Initial semen handling

Semen collection was performed during the breeding season. At all the sampling times, the ejaculate volume, sperm concentration, and total sperm output (ejaculate volume × sperm concentration) were calculated immediately after collection. Ejaculates were collected by artificial vagina at 40°C (IMV Technologies, L'Aigle, France) in the presence of a female decoy, and the tubes were maintained at 30°C before cooling. All the semen collections were carried out by the same investigator. The ejaculate volume was estimated by collecting them in Falcon^®^ type graduated semen collection tubes. Sperm concentration was assessed by a cell counter (NucleoCounter SP-100, ChemoMetec, Allerod, Denmark). Ejaculates were diluted 1:1 (v/v) with INRA 96. The samples were then refrigerated using a rate of −0.5°C/min from 30°C down to 15°C. After that, the final concentration was adjusted to 1,600 × 10^6^ sperm/ml and the samples were packed into 0.25 ml plastic straws. The seminal doses were stored at 15°C. Sperm evaluation was performed 6–8 h after semen collection.

### Ultrasonography evaluation of testicular function

For testicular evaluation function, all examinations were carried out by the same technician. Measurements were performed using the same real-time ultrasound scanner equipped with a 7.5 MHz linear array and 10 MHz high-frequency linear array transducers to evaluate the testicular echotexture and testicular blood flow, respectively.

#### Testicular echotexture

The probe was positioned by pressing on the center of the testicle transversely, and at least three clips per testicle were measured (technical replicates). The software Ecotext^®^ (HUMECO; Huesca, Spain) was used for analyzing the parenchyma echotexture. The following parameters were measured: Ecotext 1 (EC1: black pixels number), Ecotext 2 (EC2: white pixels number), Ecotext 3 (EC3: mean gray level of pixels), the tubular density (density of tubules/cm^2^), the tubular diameter [mean diameter (μm) of the lumen of the seminiferous tubules] and the tubular area [proportion (%) of the total area that was occupied by the lumen of the tubules in the parenchyma].

#### Testicular blood flow

Doppler parameters were measured in the supratesticular artery located in the spermatic cord region, and the transducer was positioned at a midway point between the inguinal ring and the testicle (34). For distinguishing between a testicular artery and vein by Doppler analysis, an artery, for example, will have a waveform on the spectral graph that reflects the arterial pulse in each cardiac cycle (systole and diastole). However, the flow in a vein is almost constant, that is, without a pulse. At least three consecutive waveforms were measured per testicle (technical replicates), and the Doppler parameters were automatically calculated by the software package provided with the ultrasound machine. Peak systolic velocity (PSV) was measured, and the Doppler indices studied were resistive index [RI = (maximum velocity-minimum velocity)/maximum velocity] and pulsatility index [PI = (maximum velocity-minimum velocity)/mean velocity]. Furthermore, the total artery blood flow (TABF) was calculated [mean velocity × *A*; *A* (cross-section of the artery): π*r*^2^]. Based on previous studies (11, 19, 35), the angle between the long axis of the vessel and the Doppler beam was from 20 to 60 degrees in the direction of the blood flow. Additionally, the Doppler gate was kept constant at 1 mm. To minimize variations in measurements, the ultrasound settings (focus, gains, brightness, and contrast) were standardized, fixed, and used equally for all examinations like others authors (9).

### Sperm evaluation

#### Motility and kinetic parameters by CASA

The motility and kinetic parameters were assessed using the Assisted Sperm Analysis (CASA) (Sperm Class Analyzer -SCA- software V 6.3.0.59; Microptic S.L., Barcelona, Spain). The parameters setting was set to capture at 100 frames/s a total of 50 frames, and particles with an area of 20–70 μm^2^ were considered compatible with the head area. Aliquots of each ejaculate were diluted to 25 × 10^6^ sperm/ml in an extender (TES-Tris-fructose and 1% egg yolk) and tempered on a 37°C plate for 5 min. After that, a 5 μl drop was placed in a Makler counting cell chamber (10 μm depth; Sefi Medical Instruments, Haifa, Israel). Samples were examined with an × 10 negative phase contrast objective in a microscope equipped with a warmed stage at 38°C (Eclipse E400, Nikon, Tokyo, Japan). At least 400 sperm from four different randomly selected fields were captured and analyzed. The reported kinetic parameters were the velocity according to the straight path (VSL, μm/s); the amplitude of the lateral displacement of the sperm head (ALH, μm); the head beat-cross frequency (BCF, Hz); the total motility (TM), defined as the percentage of sperm with VCL (curvilinear velocity) >15 μm/s; the progressive motility (PM), defined as the percentage of sperm with VCL >45 μm/s; and the rapid progressive motility (RAP PM), defined as the percentage of sperm with VCL >75 μm/s. All parameters were previously described by Palacin-Martinez et al. (36).

#### Sperm functionality by flow cytometry

##### Staining for determination of viability, caspases 3 and 7 activity, and mitochondrial functionality

Fluorescence probe Zombie Violet™ Fixable Viability Kit was acquired from BioLegend (San Diego, CA, USA), and CellEvent™ Caspase-3/7 Green Detection Reagent and CellROX™ Deep Red Reagent were supplied from ThermoFisher (Invitrogen, Eugene, Oregon, USA).

For staining, a protocol previously described by Riesco et al. (37) was used. Sperm samples were diluted in PBS to a concentration of 2 × 10^6^ sperm/ml to wash the cells by short centrifugation (15”; MiniSpin plus, Eppendorf, Hamburg, Germany) with the removal of the supernatant. Then, cells were incubated at room temperature and in the dark for 30 min with 96 μl Zombie Violet™ (1:1,000 final dilution), 2 μl CellEvent™ Caspase-3/7 (4 μM final concentration), and 2 μl CellROX™ (5 μM final concentration). After that, a new wash was performed to stop cell staining and avoid an over-staining effect, and the pellet was resuspended in 1 ml PBS, immediately conducting the analysis by flow cytometry.

The combination Zombie Violet™ Fixable Viability Kit, CellEvent™ Caspase-3/7 Green Detection Reagent, and CellROX™ Deep Red was used to simultaneously determine the viability through plasma membrane integrity, caspases 3 and 7 activity as a marker of apoptosis, and mitochondrial function through reactive oxygen species (ROS) content, respectively.

##### Flow cytometry analyses

Flow cytometry analyses were conducted in the flow cytometer MACSQuant Analyzer 10 (Miltenyi Biotech, Bergisch Gladbach, Germany) equipped with three lasers emitting at 405, 488, and 635 nm (violet, blue, and red, respectively) and 10 photomultiplier tubes. Violet fluorescence was detected in V1 (excitation 405 nm, emission 450/50 nm), green fluorescence was detected in B1 (excitation 488 nm, emission 525/50 nm), and red fluorescence was detected in R1 [excitation 635 nm, emission 655–730 nm (655 LP + split 730)]. Samples were acquired using MACS Quantify software (Miltenyi Biotech, Bergisch Gladbach, Germany), recording a total of 40,000 cells per sample at a flow rate of 200–300 cells/s. Data were analyzed using FlowJo V 10.2 (Ashland, Wilmington, DE, USA). The interest sperm subpopulations assessed were plotted as follows: viable sperm (Zombie Violet™ low intensity -alive-), apoptotic sperm (CellEvent™ Caspase 3/7 positive), and sperm with high mitochondrial activity (CellROX™ positive).

### Statistical analyses

Data were analyzed with SAS/STAT^®^ version 9.1 statistical package (SAS Institute, Cary, NC, USA). Data were analyzed by a mixed linear model (MIXED procedure), considering the male effect as a random factor. Libido was analyzed by Kruskal–Wallis test (NPAR1WAY procedure). Relations between sperm quality parameters and ultrasonography measurements were studied by Pearson's correlation. The results are displayed as the mean ± standard error of the mean (SEM). Differences were statistically significant at *P* ≤ 0.05.

## Results

### Testicular yield

**Figure 2:**
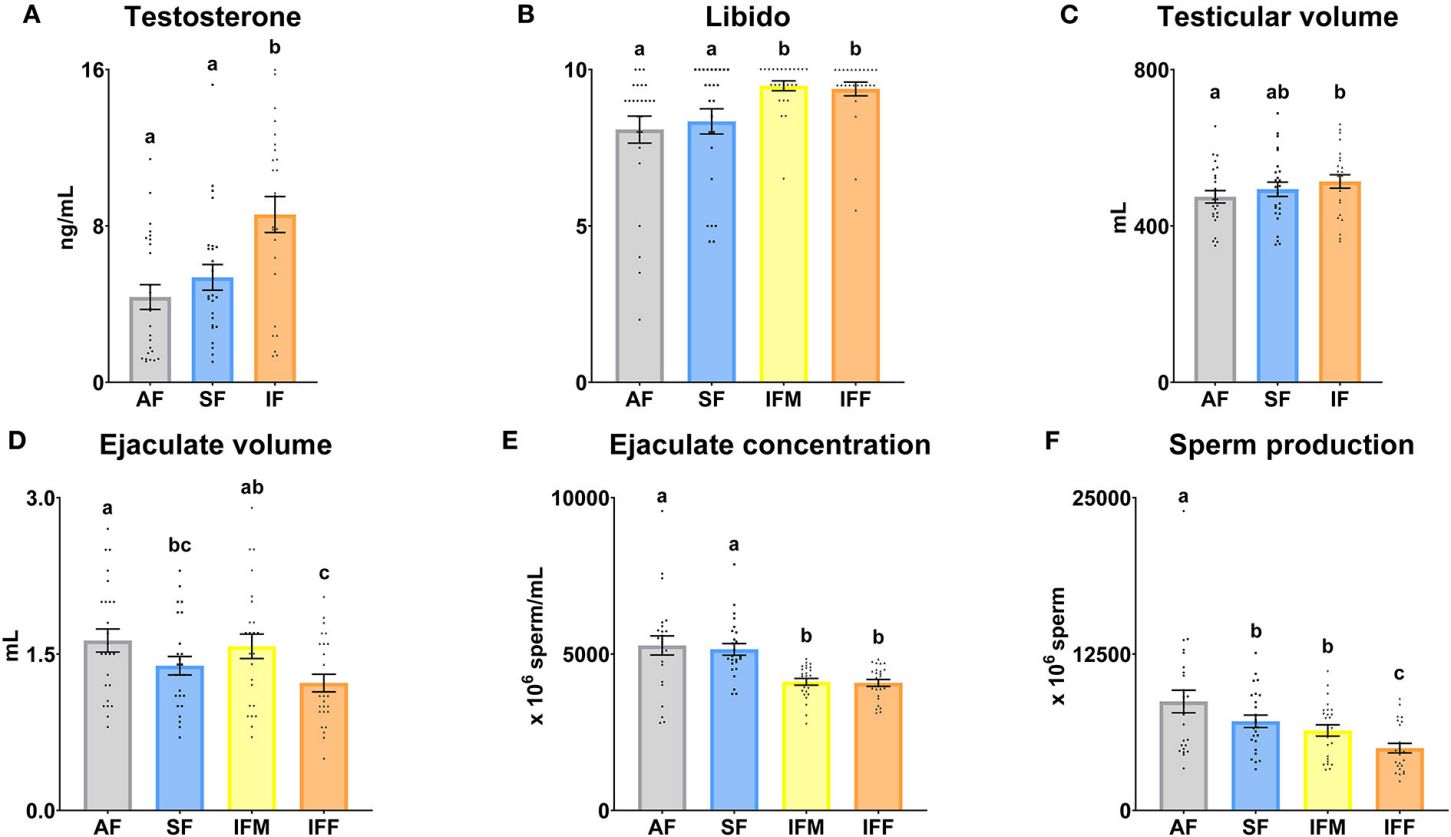
Ram reproductive values according to semen collection frequencies. **(A)** Testosterone, serum testosterone levels (ng/ml); **(B)** Libido, subjective evaluation (zero to 10); **(C)** Testicular volume (ml); **(D)** Ejaculate volume (ml); **(E)** Ejaculate concentration (× 10^6^ sperm/ml); **(F)** Sperm production (× 10^6^ sperm). The same 25 males were analyzed in each experimental group (AF, abstinence semen collection frequency; SF, standard semen collection frequency; IFM, intensive semen collection frequency on Monday; and IFF, intensive semen collection frequency on Friday). Graph dots represent individual male values [graphs **(A–C)**] and ejaculates [graphs **(D–F)**]. Means (±SEM) are shown. Different lowercase superscripts letters (a–c) indicate differences (*P* ≤ 0.05) among the semen collection frequencies.

Significant differences (*P* ≤ 0.05) were found among all the frequencies of semen collection in the serum testosterone levels (Figure 2A), with the highest levels in the IF. Libido was significantly (*P* ≤ 0.05) higher in intensive semen collections (IFM and IFF; Figure 2B) compared to AF and SF. Concerning testicular measurements, a significant increase in volume was observed in IF compared to AF (*P* ≤ 0.05; Figure 2C). According to sperm production, the ejaculate volume was significantly decreased (*P* ≤ 0.05) in SF and IFF in comparison with AF, but non-significant differences (*P* > 0.05) between both were found (Figure 2D). Sperm concentration decreased in both sperm evaluations of IF (Figure 2E), and sperm production was gradually lower (*P* ≤ 0.05) with increasing intensity of semen collection frequency. However, non-significant differences concerning sperm production were revealed between SF and IFM (*P* > 0.05; Figure 2F).

### Ultrasonography evaluation of testicular function

Right and left testis did not show significant differences with respect to echotexture and Doppler parameters (data not shown). Thus, the means of the right and lefts testis were used for further analysis.

**Figure 3:**
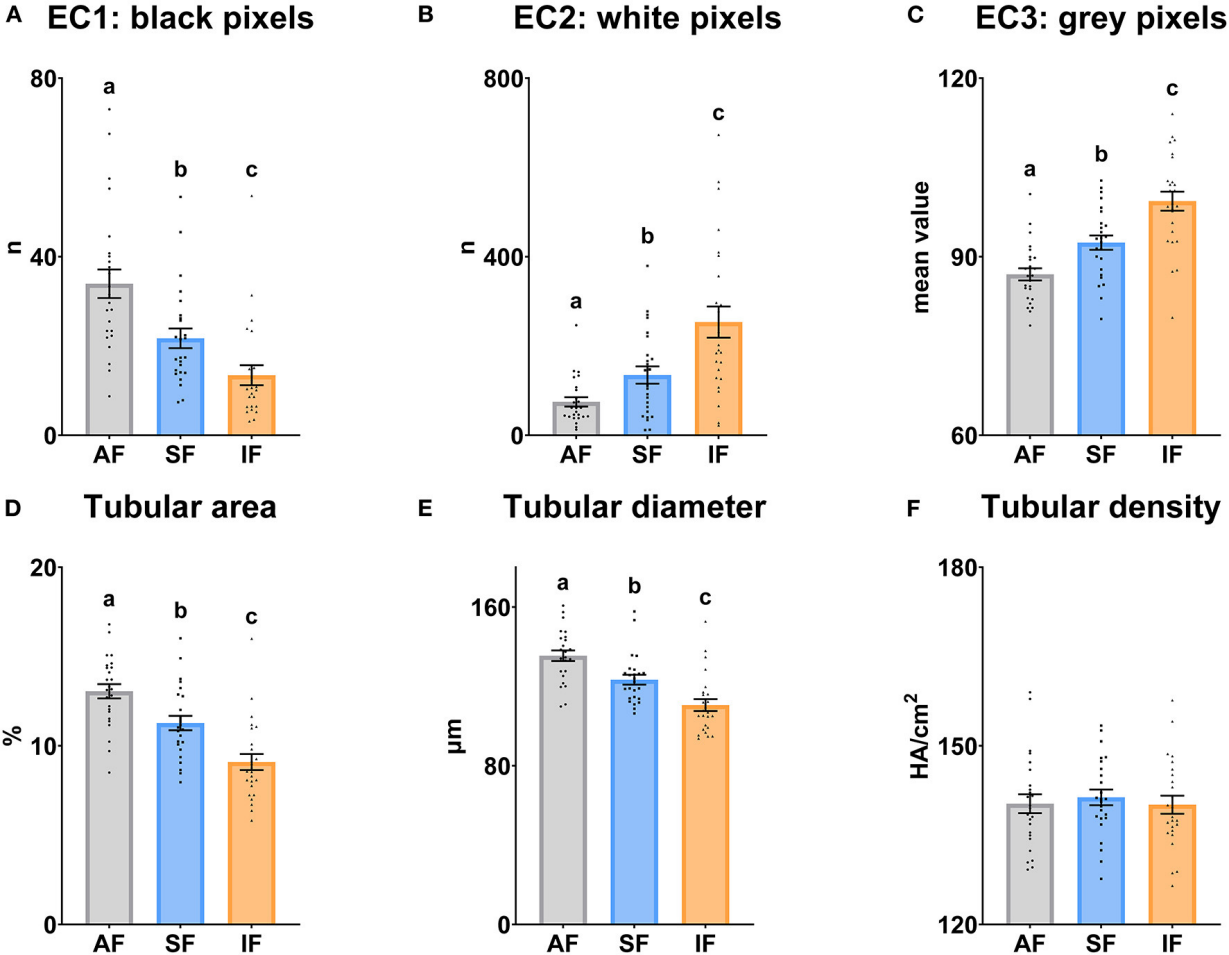
Echotexture characteristics of the ram testes according to semen collection frequencies. **(A)** EC1, Ecotext 1 (black pixels); **(B)** EC2, Ecotext 2 (white pixels); **(C)** EC3, Ecotext 3 (mean value of gray pixels); **(D)** Tubular area, proportion (%) of the total area corresponding with the lumen of the seminiferous tubules; **(E)** Tubular diameter, mean diameter (μm) of the lumen of the seminiferous tubules; **(F)** Tubular density, the density of hypoechogenic areas per cm^2^ corresponding with the seminiferous tubules. The same 25 males were analyzed in each experimental group (AF, abstinence semen collection frequency; SF, standard semen collection frequency; IF, intensive semen collection frequency). Graph dots represent individual male values. Means (±SEM) are shown. Different lowercase superscripts letters (a–c) indicate differences (*P* ≤ 0.05) among the semen collection frequencies.

The testicular echotexture results are shown in Figure 3. EC1, tubular area and tubular diameter decreased significantly (*P* ≤ 0.05) when the frequency of semen collection increased. However, EC2 and EC3 increased significantly (*P* ≤ 0.05) under the same conditions. The tubular density was similar among the semen collection frequencies (*P* > 0.05).

**Figure 4:**
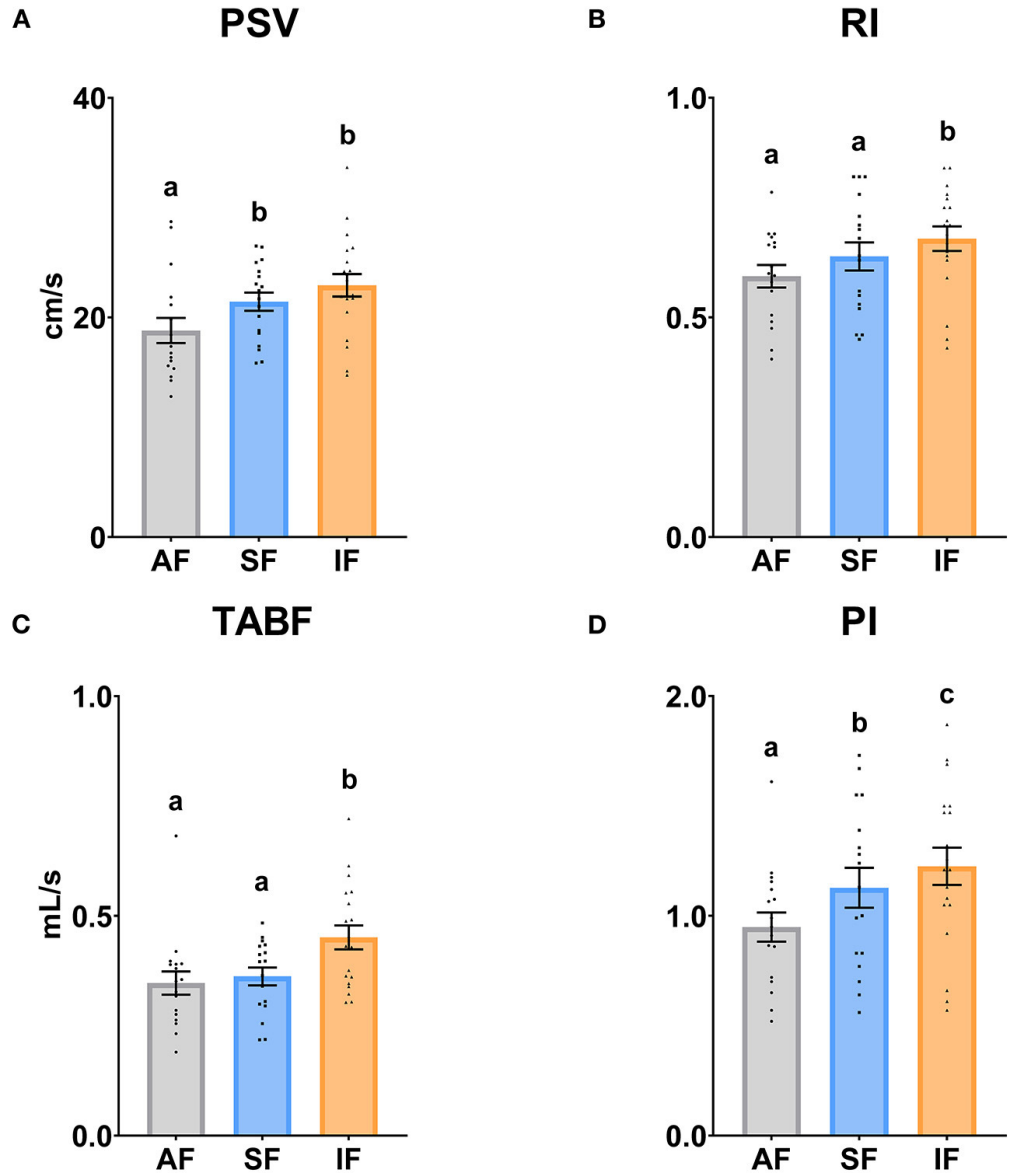
Ram supratesticular artery Doppler parameters according to semen collection frequencies. **(A)** PSV, peak systolic velocity (cm/s); **(B)** RI, resistive index; **(C)** TABF, total artery blood flow (ml/min); **(D)** PI, pulsatility index. The same 17 males were analyzed in each experimental group (AF, abstinence semen collection frequency; SF, standard semen collection frequency; IF, intensive semen collection frequency). Graph dots represent individual male values. Means (±SEM) are shown. Different lowercase superscripts letters (a–c) indicate differences (*P* ≤ 0.05) among the semen collection frequencies.

**Figure 5:**
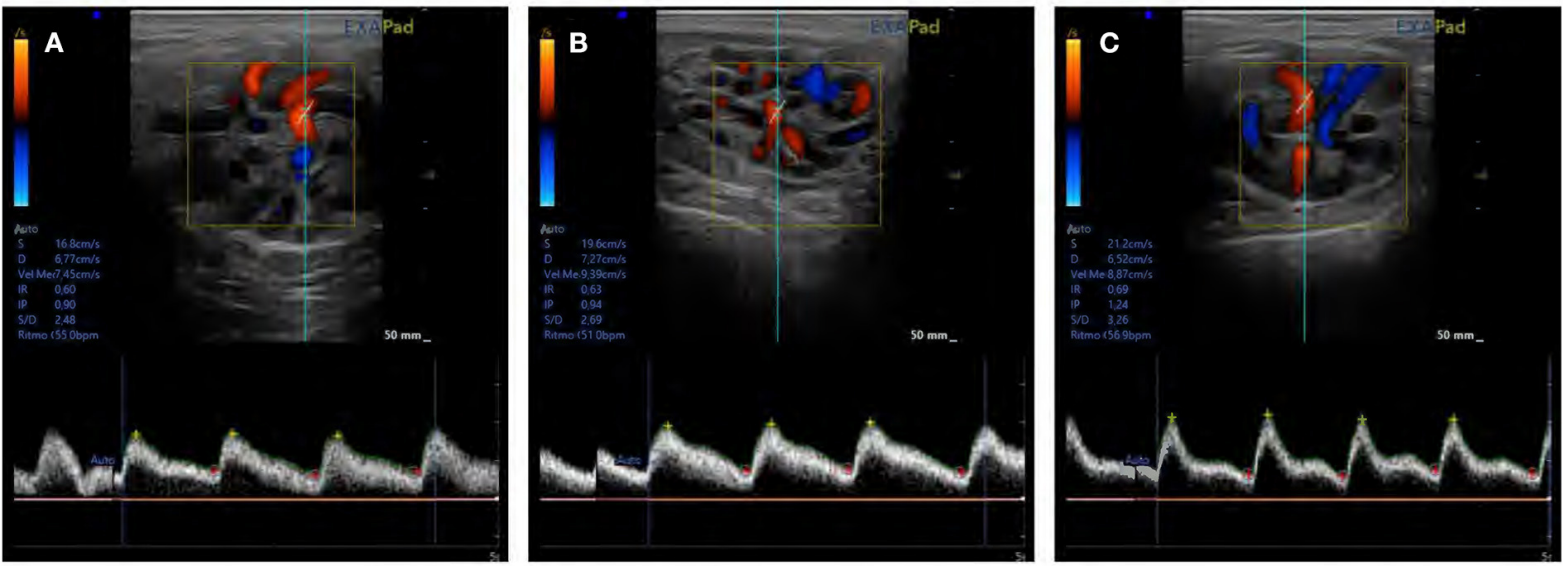
An assessment of testicular blood flow in supratesticular artery using pulse Doppler ultrasonography in **(A)** abstinence, **(B)** standard, and **(C)** intensive semen collection frequencies.

The testicular vascularization results are shown in Figures 4, 5. The PSV parameter was significantly higher (*P* ≤ 0.05) in SF and IF with respect to AF. In relation to RI and TABF parameters, both increased significantly (*P* ≤ 0.05) in IF in comparison to AF and SF. In addition, there were significant differences among all semen collection frequencies in PI (*P* ≤ 0.05).

### Sperm quality evaluation

#### Motility and kinetic parameters

**Figure 6:**
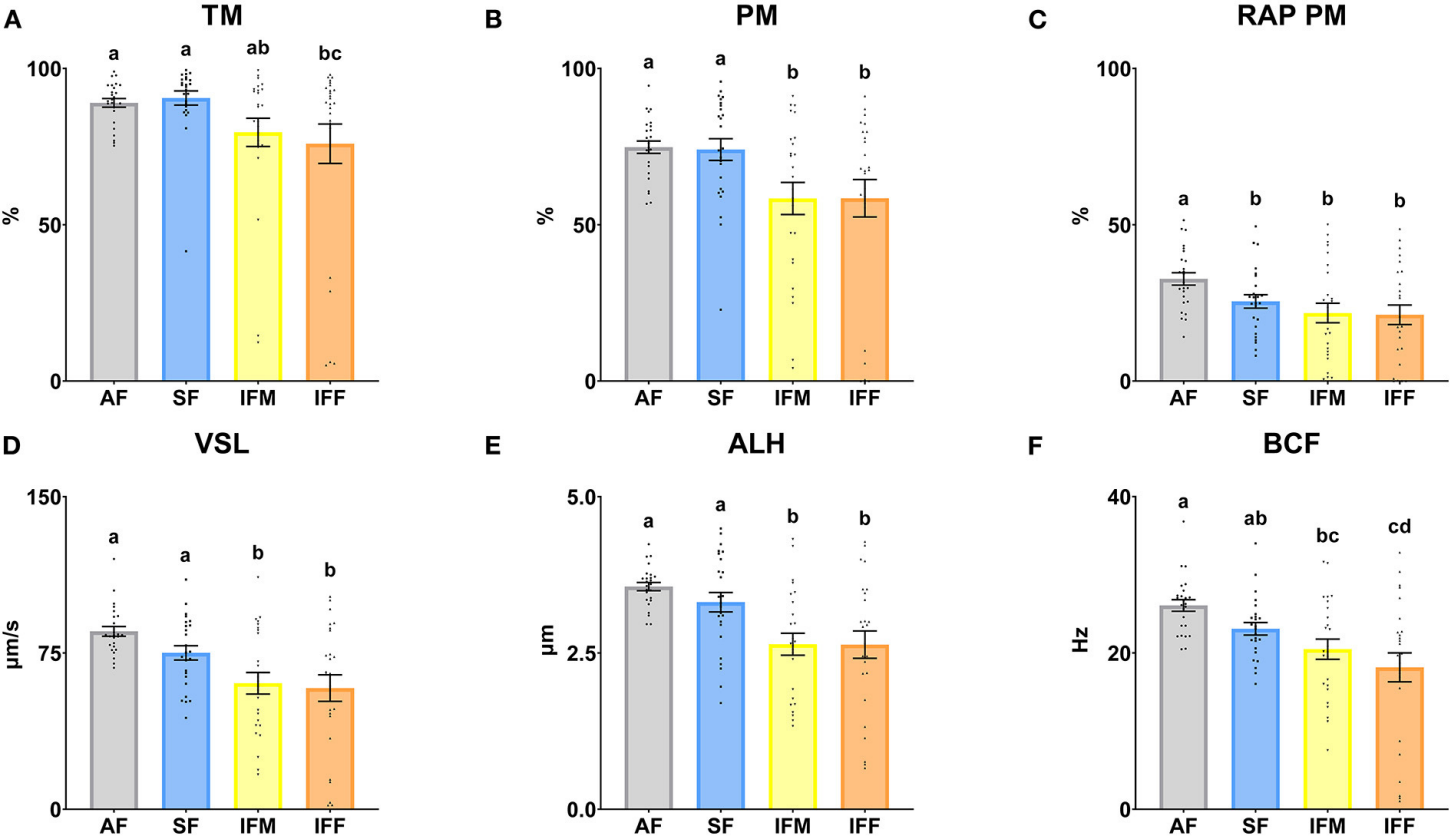
Ram sperm motility according to ejaculate collection frequencies. **(A)** TM, total motility (%); **(B)** PM, progressive motility (%); **(C)** RAP PM, rapid progressive motility (%); **(D)** VSL, straight-line velocity (μm/s); **(E)** ALH, head lateral amplitude (μm); **(F)** BCF, beat frequency (Hz). The same 25 males were analyzed in each experimental group (AF, abstinence semen collection frequency; SF, standard semen collection frequency; IFM, intensive semen collection frequency on Monday; and IFF, intensive semen collection frequency on Friday). Graph dots represent individual male values. Means (±SEM) are shown. Different lowercase superscripts letters (a–d) indicate differences (*P* ≤ 0.05) among the semen collection frequencies.

The sperm motility parameters are shown in Figure 6. There were significant differences between AF and IFF in TM (*P* ≤ 0.05), decreasing when the semen collection intensity increased. In the same way, the TM was significantly higher (*P* ≤ 0.05) in SF in comparison with IFF (Figure 6A). Regarding RAP PM, the highest percentage was observed in AF (*P* ≤ 0.05; Figure 6C). PM, VSL, and ALH were significantly higher (*P* ≤ 0.05) in AF and SF in comparison with IFM and IFF (Figures 6B,D,E). BCF was significantly higher (*P* ≤ 0.05) in AF in comparison with IFM and IFF. In the same way, SF was significantly higher (*P* ≤ 0.05) than IFF (Figure 6F).

#### Sperm functionality

**Figure 7:**
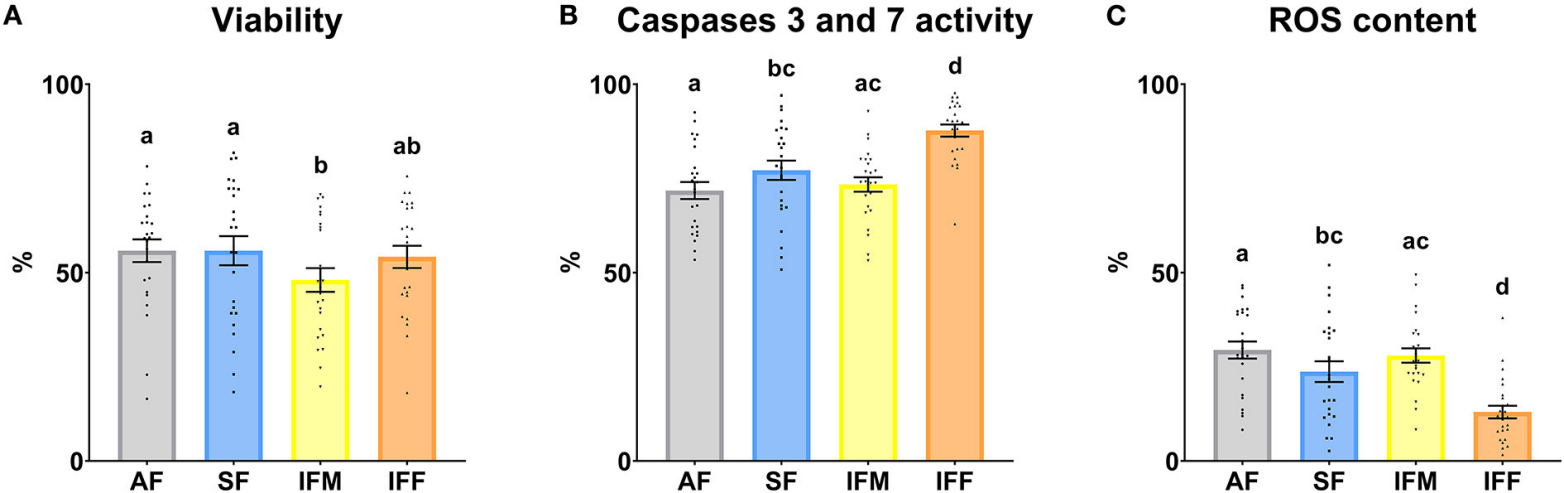
Ram sperm functionality according to ejaculate collection frequencies. **(A)** Viability, Zombie Violet™ (%); **(B)** Caspases 3 and 7 activity, CellEvent™ Caspase-3/7 Green (%); **(C)** ROS content, CellROX™ Deep Red (%). The same 25 males were analyzed in each experimental group (AF, abstinence semen collection frequency; SF, standard semen collection frequency; IFM, intensive semen collection frequency on Monday; and IFF, intensive semen collection frequency on Friday). Graph dots represent individual male values. Means (±SEM) are shown. Different lowercase superscripts letters (a–d) indicate differences (*P* ≤ 0.05) among the semen collection frequencies.

Attending to the flow cytometry analysis, total viability was significantly lower (*P* ≤ 0.05) in IFM with respect to AF and SF (Figure 7A). The percentage of apoptotic sperm, with active caspases 3 and 7, was significantly higher (*P* ≤ 0.05) in IFF compared with the other experimental groups. Moreover, this parameter also was significantly higher (*P* ≤ 0.05) in SF with respect to AF (Figure 7B). Finally, the percentage of sperm with high mitochondrial activity showed the same statistically significant differences as the previous parameter analyzed (Figure 7C) but with an opposite trend.

### Correlations between ultrasonography parameters and sperm quality

**Figure 8:**
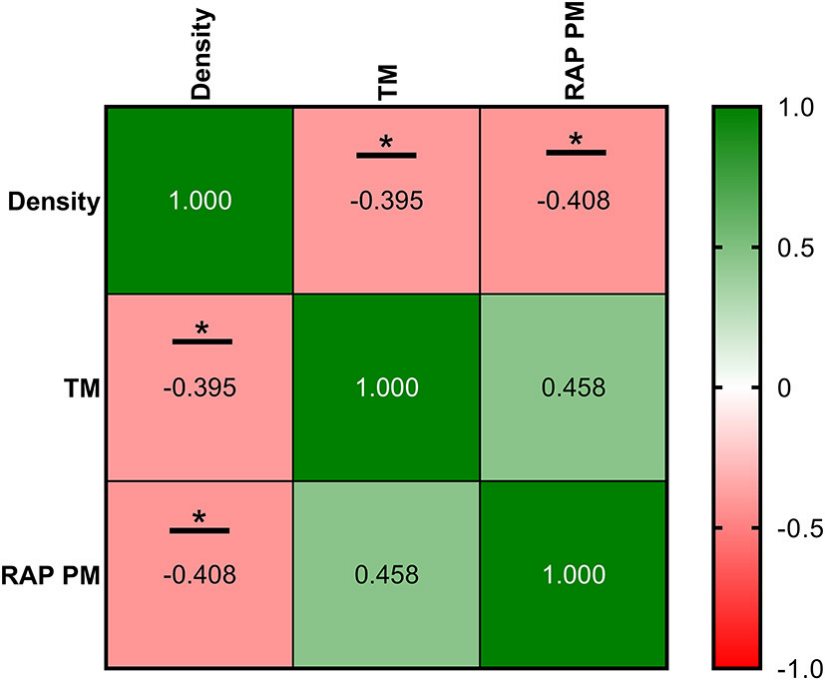
Correlation coefficients between ultrasonography measurements and sperm quality parameters in abstinence semen collection frequency (AF). Density, the density of hypoechogenic areas per cm^2^ corresponding with the seminiferous tubules; TM, total motility (%); RAP PM, rapid progressive motility (%). The same 25 males were analyzed in each parameter. The *R* squared value between two parameters is represented in each cell and graph. In the correlation matrix, green color indicates positive correlations, and red color indicates negative relationships. The color intensity represents the strength of the correlation between two parameters. Asterisks show significant correlations (*P* ≤ 0.05) between ultrasonography measurements and sperm quality parameters.

**Figure 9:**
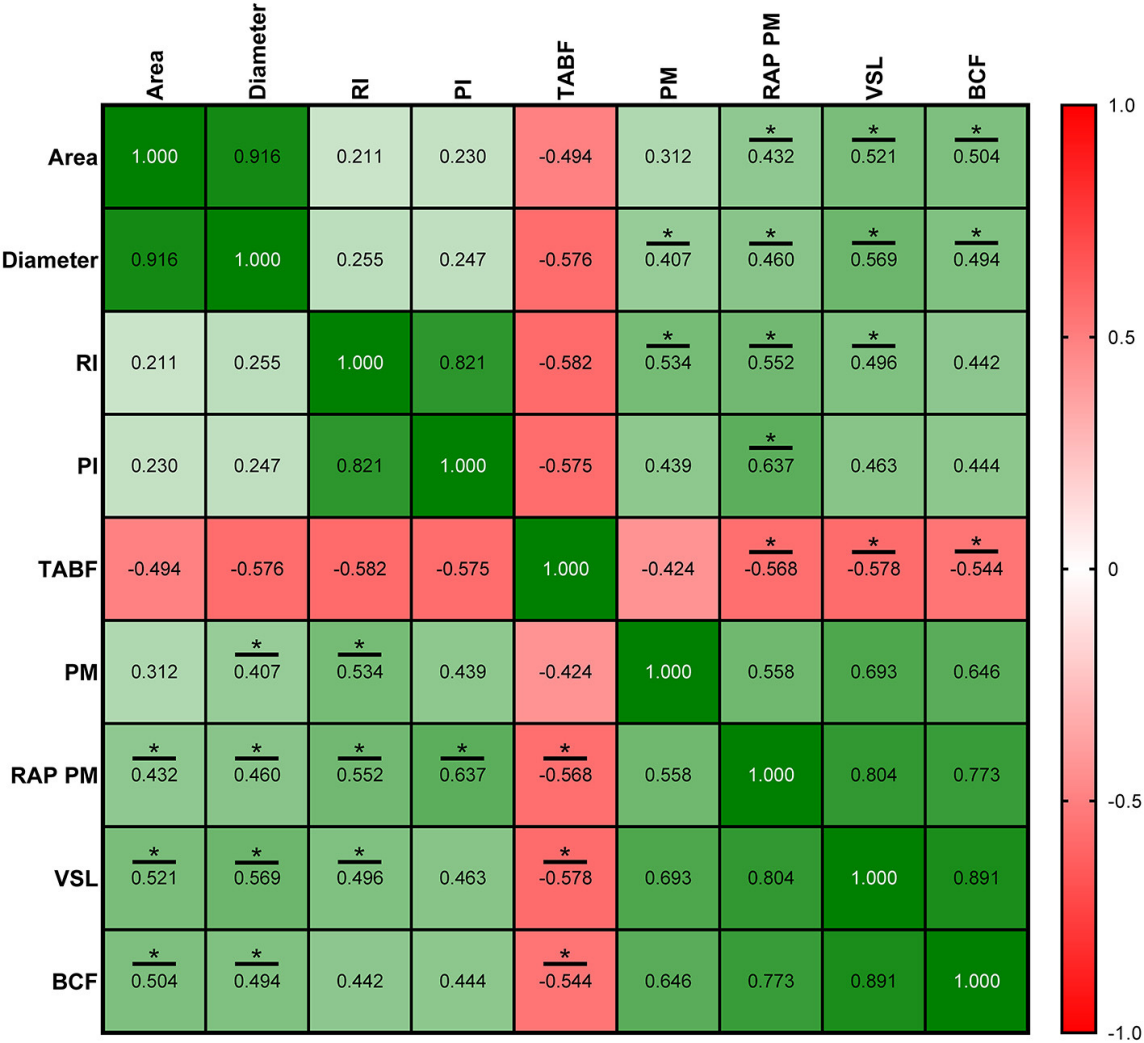
Correlation coefficients between ultrasonography measurements and sperm quality parameters in standard semen collection frequency (SF). Area, proportion (%) of the total area corresponding with the lumen of the seminiferous tubules; Diameter, mean diameter (μm) of the lumen of the seminiferous tubules; RI, resistive index; PI, pulsatility index; TABF, total artery blood flow (ml/min); PM, progressive motility (%); RAP PM, rapid progressive motility (%); VSL, straight-line velocity (μm/s); BCF, beat frequency (Hz). The same 25 males were analyzed in each parameter. The *R* squared value between two parameters is represented in each cell and graph. In the correlation matrix, green color indicates positive correlations, and red color indicates negative relationships. The color intensity represents the strength of the correlation between two parameters. Asterisks show significant correlations (*P* ≤ 0.05) between ultrasonography measurements and sperm quality parameters.

**Figure 10:**
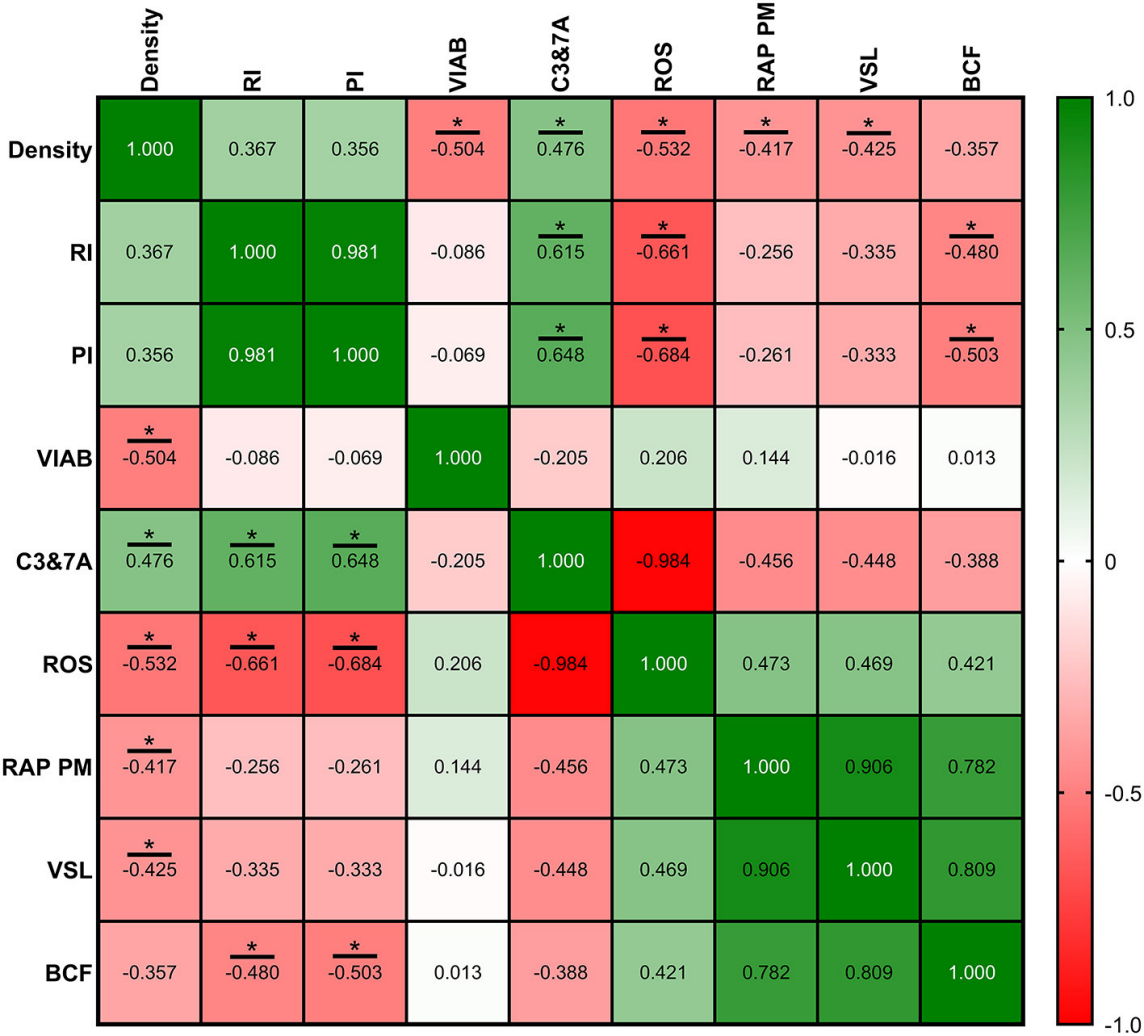
Correlation coefficients between ultrasonography measurements and sperm quality parameters in intensive semen collection frequency (IF). Sperm quality parameters used were measured on Friday (corresponding with IRF). Density, density of hypoechogenic areas per cm^2^ corresponding with the seminiferous tubules; RI, resistive index; PI, pulsatility index; VIAB, viability (%); C3&7A, caspases 3 and 7 activity (%); ROS, ROS content (%); RAP PM, rapid progressive motility (%); VSL, straight-line velocity (μm/s); BCF, beat frequency (Hz). The same 25 males were analyzed in each parameter. The *R* squared value between two parameters is represented in each cell and graph. In the correlation matrix, green color indicates positive correlations, and red color indicates negative relationships. The color intensity represents the strength of the correlation between two parameters. Asterisks show significant correlations (*P* ≤ 0.05) between ultrasonography measurements and sperm quality parameters.

Correlations between ultrasonography parameters and sperm quality are shown in Figures 8–10, related to AF, SF, and IF, respectively. The highest correlation between ultrasonography and sperm quality in AF was found between Tubular density and RAP PM (*R*^2^ = −0.408; Figure 8). Concerning SF, the pulsatility index presented the highest correlation with RAP PM (*R*^2^ = 0.637). Also, RI, TABF, Area, and Diameter showed significant moderate positive correlations with motility parameters (Figure 9). Attending to IF, Doppler indexes (RI and PI) correlated strongly and positively with C3&7A. However, the same Doppler indexes showed strong negative correlations with ROS (*P* ≤ 0.05). All the correlations studied are included in a correlation matrix (Figure 10).

### Field results: Semen yield, sperm quality and fertility trials

**Figure 11:**
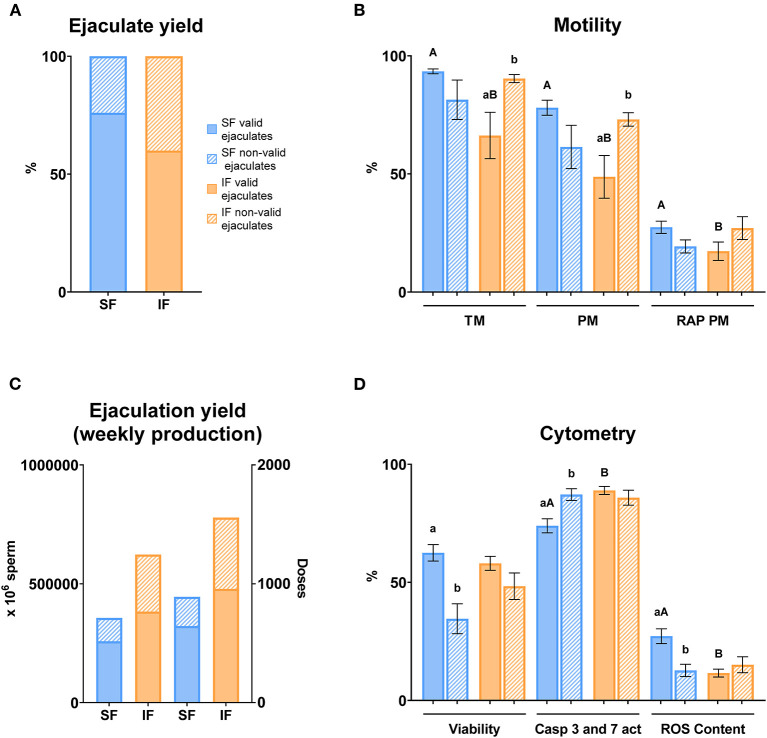
Semen yield and sperm quality in valid and non-valid ejaculates. Criteria of valid ejaculate: ejaculate volume >0.5 ml, mass motility >3, sperm concentration > 3,000 × 10^6^ sperm/ml. Non-valid ejaculates: some of the criteria previously described below the minimum value. **(A)** Ejaculate yield: % of valid and non-valid ejaculates in two semen collection frequencies (SF, standard semen collection frequency; IF, intensive semen collection frequency corresponding with IFF; Friday); **(B)** Motility: sperm samples of SF (blue columns) and IF (orange columns); TM, total motility (%); PM, progressive motility (%); RAP PM, rapid progressive motility (%); **(C)** Ejaculation yield (weekly production): number of sperm and doses produced weekly in two semen collection frequencies (SF and IF); **(D)** Cytometry: sperm samples of SF (blue columns) and IF (orange columns); Viability, Zombie Violet™ (%); Caspases 3 and 7 activity, CellEvent™ Caspase-3/7 Green (%); ROS content, CellROX™ Deep Red (%). The same 25 males were analyzed in each experimental group. Different lowercase superscripts letters (a,b) indicate differences (*P* ≤ 0.05) for each assessment point between valid and non-valid ejaculates. Different lowercase superscripts capital letters (A,B) indicate differences (*P* ≤ 0.05) for each assessment point between semen collection frequencies.

Concerning ejaculate yield, a higher percentage of valid ejaculates was recorded in SF (76%) compared with IF (60%) (Figure 11A). In terms of ejaculation yield, the percentage of valid sperm numbers and doses were higher in SF in comparison with IF (Figure 11C). Motility parameters registered significant differences (*P* ≤ 0.05) between SF and IF in valid ejaculates in terms of TM, PM, and RAP PM, being lower in IF. On the other hand, significant differences were found between valid and non-valid ejaculates in IF with respect to TM and PM (*P* ≤ 0.05; Figure 11B). Cytometry analyses showed that apoptosis was significantly lower in valid ejaculates in SF compared to valid ejaculates in IF (*P* ≤ 0.05). Contrary to this, mitochondrial activity was higher in valid ejaculates in SF with respect to valid ejaculates in IF (*P* ≤ 0.05). Regarding the valid ejaculates comparison, there were significant differences (*P* ≤ 0.05) in viability, apoptosis, and mitochondrial activity in SF (Figure 11D).

**Figure 12:**
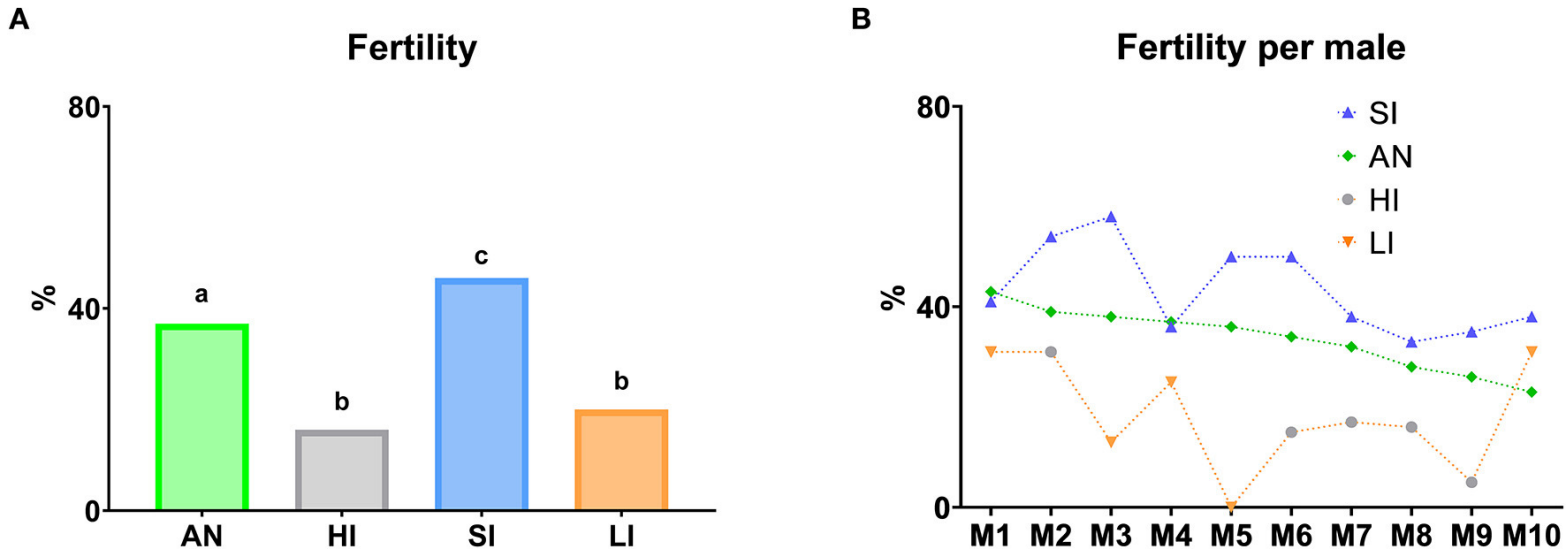
Fertility according to different semen collection frequencies. **(A)** Fertility per group according to abstinence days before semen collection for AI. **(B)** Descriptive study of the fertility per male according to abstinence days before semen collection for AI. AN (Annual fertility), annual average of lambing ewes/inseminated ewes (%); HI (Fertility high interval), fertility with seminal doses obtained from males with at least 10 abstinence days; SI (Fertility standard interval), fertility with seminal doses obtained from males with two to five abstinence days; LI (Fertility low interval), fertility with seminal doses obtained from males with semen collection the previous day. In graphic **(A)**, different lowercase superscripts letters (a–c) indicate differences (*P* ≤ 0.05) for each assessment point among semen collection ratios due to abstinence days before semen collection for AI. In graphic **(B)**, graph dots represent individual males.

A fertility trial was carried out considering an abstinence period before semen collection for AI (Figure 12A). According to this, the highest fertility rates were obtained with SI of semen collection (*P* ≤ 0.05). However, when males were submitted both high or low intervals (HI and LI, respectively), fertility rates decreased significantly (*P* ≤ 0.05), following the same trend with respect to SI and AN. Additionally, there was no identification of differences between them. In addition, the annual fertility was significantly lower than SI (*P* ≤ 0.05). With respect to the descriptive study per male, we observed the same trend in most of them.

According to this, most males presented higher fertility rates with SI of semen collection. However, when males were submitted both high or low intervals (HI or LI, respectively), fertility rates decreased following the same trend (Figure 12B).

## Discussion

The improvement of semen donor rams in terms of sperm quality and yield in reproduction centers is a feasible alternative to increase the results of artificial insemination (AI). In this context, semen collection frequency is a common factor studied in several species such as ram (38), boar (39), stallion (40), pigeon (41) or human (42). In our study, sperm production and quality parameters decreased when the semen collection frequency increased. These evidences are in accordance with findings from other studies carried out in rams (6) or humans (43), where it was also reported that increased semen collection frequency had a negative impact on sperm count. Our current findings were consistent with those in a report from Ollero et al. (5), where about 27% less ejaculate volume was obtained after 1 or 2 days of abstinence than after abstinence for 3 days, and sperm concentration decreased significantly as the abstinence period decreased. In our study, 72 h of sexual abstinence (weekend) were enough for a partial recuperation of ejaculate volume and sperm production, allowing us to discard a loss of testicular functionality phenomenon. Linked to our findings on sperm quantity, sperm motility, which is used in the routine evaluation of sperm (5), showed a significant (*P* ≤ 0.05) decrease in IF, demonstrating the influence of semen collection frequency in sperm motility parameters. Our findings in ovine species are consistent with those reported for rams (6) and boars (39). Concerning the sperm functionality, there were no significant differences (*P* > 0.05) in viability. In contrast, more advanced cytometry parameters in sperm preservation showed interesting changes. Caspases 3 and 7 activity presented the highest value in IF. These caspases are specific cysteinyl aspartate proteases that execute the breakdown of structural proteins and DNA (44). For this reason, this finding was related to apoptotic changes, which could compromise the ability to fertilize the oocyte (45, 46). Moreover, the lowest percentage of ROS content was found in IF. The ROS content measured by the CellROX probe in sperm mainly reflects intense mitochondrial activity rather than oxidative stress (47–50). Thus, a high frequency of semen collection could reduce the mitochondrial activity of ram sperm. Contrary to our findings, cytometry parameters in humans were not significantly affected after a 2-week period of daily ejaculation, although a decreasing trend in intracellular ROS production was also observed (43). More interestingly, after a short recovery period (3 days of abstinence during the weekend) in the IF model, apoptosis and mitochondrial activity were significantly improved. The current findings obtained in sperm quality are in accordance with Ihukwumere and Okere observations (51) and could occur because sperm cells may need a minimum storage time in the cauda epididymis. Several epididymal components have collaborated in fertilizing capacity and motility of sperm cells due to biochemical and biophysical changes and interactions (52). For instance, clusterin, which is the most abundant protein of the cauda epididymal fluid in rams (53), participates in sperm maturation, lipid transport (54), and sperm membrane remodeling; acts as chaperone (55); and prevents peroxidative damage (56).

Traditional methods such as libido and clinical examinations (3, 7, 8) or basic ultrasound evaluation including testicular volume (26) have been used to complete the BSE. In our study, a testicular volume increase (*P* ≤ 0.05) was detected in IF in comparison with AF, which could be explained by a high demand for sperm production that provokes a temporal and compensatory testicular hypertrophy (57). Another possible partial explanation to the observed increase of testicular volume is the advance of the breeding season (9) since the duration of the experiment was 2 months. As expected, serum testosterone and libido were higher (*P* ≤ 0.05) in IF in relation to the other experimental groups. This could be related to the season and the testicular overexertion, which could trigger the activation of different pathways of the hypothalamus-hypophysis axis, provoking the testosterone increase. Some authors demonstrated this effect when they applied several treatments to improve the reproduction performance, such as buserelin (58) or eCG (59).

In these types of studies, new parameters and integrative studies on ultrasonography assessment could be more predictive and reinforce the optimization of the current ram reproductive handling to obtain high sperm quality and fertility. Consequently, we performed a testicular echotexture test using Ecotext^®^ and a testicular vascularization evaluation using Doppler parameters in combination with some sperm quality analyses, including motility and sperm physiology, to analyze their possible correlations. According to the published literature, this is the first time that a male factor (frequency of semen collection) was used in an integrative way within the ram BSE: from ultrasonographic evaluation, such as ram reproductive ability predictor, to measure to sperm quality analyses.

The echotexture parameters revealed changes in the parenchyma structure, increasing echogenicity as the frequency of semen collection intensifies. We observed a significant decrease in black pixels number (EC1), tubular area, and tubular diameter with the intensification of semen collection; all of them could be related to the lumen of seminiferous tubules. We also observed a significant increase in white pixels number (EC2) and mean gray level of pixels (EC3); both could be due to different lumen cell types, which was demonstrated by Giffin et al. (25). These researchers correlated the testicular echotextural attributes with the predominant cell type (the lower echotexture with the higher cell differentiation degree) in the lumen of the seminiferous tubules in the ovine species. Thus, our findings could be explained by the alteration suffered in the composition of the lumen cells under different semen collection frequencies. Moreover, in a study conducted by Camela et al. (26), peripubertal rams showed lower seminiferous tubules lumen and, therefore, greater testicular echotexture than postpubertal rams. These findings in peripubertal rams could be in accordance with the echotextural changes when the intensity of the semen collection frequency increased, showing less hypoechogenic areas related to seminiferous tubules lumen. On the other hand, the density of hypoechogenic areas per cm^2^ did not show changes among regimes. This could be because, in adult males, the relative seminiferous tubule quantity remains stable when males achieve sexual maturity (60). In spite of this, in AF, negative correlations were found between Density and TM and RAP PM. This could suggest that males with more seminiferous tubules have poor sperm motility in an abstinence semen collection frequency. Although a large amount of sperm could be stored in the epididymis in males with more seminiferous tubules, during prolonged abstinence periods, sperm are exposed to several sperm motility inhibiting factors [acidic pH and a high potassium to sodium ratio in epididymal fluid (61, 62)], which may negatively impact their future motility after ejaculation (63).

Following the integral assessment of the reproductive capacity of rams based on ultrasound evaluation, the Pulse-Doppler mode was described as an indicator of testicular functionality in standard conditions in ram (9, 10), dog (14), stallion (32), or human (64). However, Doppler parameters had not been investigated in different semen collection frequencies correlating these analyses with sperm quality assays. Firstly, PSV and PI increased significantly (*P* ≤ 0.05), and RI and TABF did not vary significantly (*P* > 0.05) in SF compared to AF. This could be explained due to PSV and PI may be early predictors of testicular blood perfusion changes, as Jolly et al. (65) described. Moreover, RI is altered when more severe disorders occur (64). Infertile dogs had lower PSV than fertile dogs without varying RI because vascular bed resistance depends on multiple factors such as diameter and tortuosity of the vessels (66). Although it has not been previously described, we observed positive correlations between some ultrasonography (Area, Diameter, RI, and PI) and sperm motility parameters (PM, RAP PM, VSL, and BCF). In this sense, other sperm quality parameters such as live sperm and sperm concentration correlated positively with some seminal plasma antioxidants such as SOD, GPx, and GSH (67). Moreover, Hedia et al. (67) confirmed positive correlations between seminal plasma antioxidants presented in high-quality spermatozoa samples and Doppler indexes, connecting with our positive correlations in SF. On the other hand, all indexes (RI, PI, and TABF) increased significantly in IF with respect to SF. This finding could indicate an increase in resistance to blood flow, pulsatility in the oscillations of the waveform, and blood flow per minute as a consequence of the testicular stress by the intensive semen collection frequency. Low oxygen tension in the seminiferous tubules is essential for spermatogenesis (68); thus, the poor-quality sperm in IF could be justified by increased blood flow with higher oxygen tension. Recently, Ntemka et al. (29) correlated Doppler indexes (RI and PI) negatively with sperm abnormalities. In our work, we found, for the first time, that these Doppler indexes correlated negatively with functionality sperm parameters measured by flow cytometry in consonance with Hedia et al. (9) and Ntemka et al. (29). Nevertheless, to find these correlations, we hypothesized that it is necessary to overexert the testis. A recent study carried out by Brito et al. (69) comparing young and senile dogs revealed a lesser sperm quality in senile dogs and did not detect significant differences in ultrasonographic B-mode evaluation (70). In this sense, vascular characteristics of the testes may represent the causal factors underlying changes in spermatogenesis and, as a consequence, affecting the sperm quality of donor rams negatively in an intensive semen collection frequency. Based on our results, sophisticated studies of testicular echotexture and vascular evaluation measured by specific software and Doppler mode, respectively, are crucial in the reproductive ultrasonography evaluation of males.

In the second part of the paper, we carried out a field trial to demonstrate the importance of semen collection frequencies in AI success. It has been demonstrated that fertility is affected by many factors (intrinsic and extrinsic) related to the female such as the age of the ewe, the lambing-AI interval, or the cumulative number of AI/ewe; the farm such as environmental conditions, the sanitary status, or reproductive handling; the technique itself; and the male such as seasonality, sperm quality, or sperm conservation (71–73). Within the male factors, the frequency of semen collection has not been previously related to fertility rates. Although the ejaculates were considered valid according to the criteria of the reproduction centers (33), when we carried out more advanced analyses such as sperm motility and functionality, we detected significant differences in several parameters (TM, PM, RAP PM, Caspases 3 and 7 activity, and ROS content) and, therefore, we think that the fertility could be altered. In our field results, the standard interval of sexual abstinence, from 2 to 5 days, represented the highest fertility rates. Moreover, in a descriptive assay, we could observe the same trend in fertility rates in most evaluated males comparing different abstinence days before semen collection for AI. As we mentioned before, this fact could be due to the influence of the storage time in the cauda epididymis (53). This work paves the way to know what would be the optimal frequency of semen collection for each ram. It would be very beneficial to group males into different frequencies of semen collection to obtain the maximum reproductive performance from each ram and increase their fertility. To achieve this, a complex ultrasonographic evaluation should be included in the ram's BSE to predict the individual ram's reproductive capacity and optimize the reproductive handling of males.

## Data availability statement

The raw data supporting the conclusions of this article will be made available by the authors, without undue reservation.

## Author contributions

RM-G: conceptualization, methodology, formal analysis, investigation, writing—original draft, data curation, and visualization. MR: conceptualization, methodology, formal analysis, investigation, supervision, data curation, writing—review and editing, and visualization. LA-L and MA: conceptualization, methodology, investigation, resources, data curation, writing—review and editing, visualization, and funding acquisition. MN-M, CP-M, CO-F, and MH: conceptualization, methodology, and investigation. PP: formal analysis, investigation, resources, data curation, writing—review and editing, visualization, supervision, and funding acquisition. LA: conceptualization, resources, data curation, writing—review and editing, visualization, supervision, project administration, and funding acquisition. All authors contributed to the article and approved the submitted version.

## Funding

This work was supported in part by Ministerio de Ciencia e Innovación (PID2021-122470OB-I00), MINECO (AGL2017-83098-R) and Junta de Castilla y León (LE253P18). RM-G was supported by Junta de Castilla y León (through the Consejería de Educación and FSE PO 1420 – CyL, fellowship ORDEN EDU/556/2019), MN-M was supported by MEC (fellowship FPU17/04142), and CP-M was supported by MINECO (PRE2018-086400).

## Conflict of interest

The authors declare that the research was conducted in the absence of any commercial or financial relationships that could be construed as a potential conflict of interest.

## Publisher's note

All claims expressed in this article are solely those of the authors and do not necessarily represent those of their affiliated organizations, or those of the publisher, the editors and the reviewers. Any product that may be evaluated in this article, or claim that may be made by its manufacturer, is not guaranteed or endorsed by the publisher.
